# Supplementary material for: Astrocyte-induced internal state transitions reshape brainwide sensory, integrative, and motor computations
Source: bioRxiv. 2026 Feb 5:2026.02.05.704034. Preprint. [Version 1] doi: 10.64898/2026.02.05.704034 (PMC12889612; doi:10.64898/2026.02.05.704034)
Supplement: 1 [file NIHPP2026.02.05.704034v1-supplement-1.pdf]

bioRxiv preprint doi: https://doi.org/10.64898/2026.02.05.704034; this version posted February 5, 2026. The copyright holder for this preprint (which was not certified by peer review) is the author/funder, who has granted bioRxiv a license to display the preprint in perpetuity. It is made available under aCC-BY 4.0 International license.

Supplemental Figures

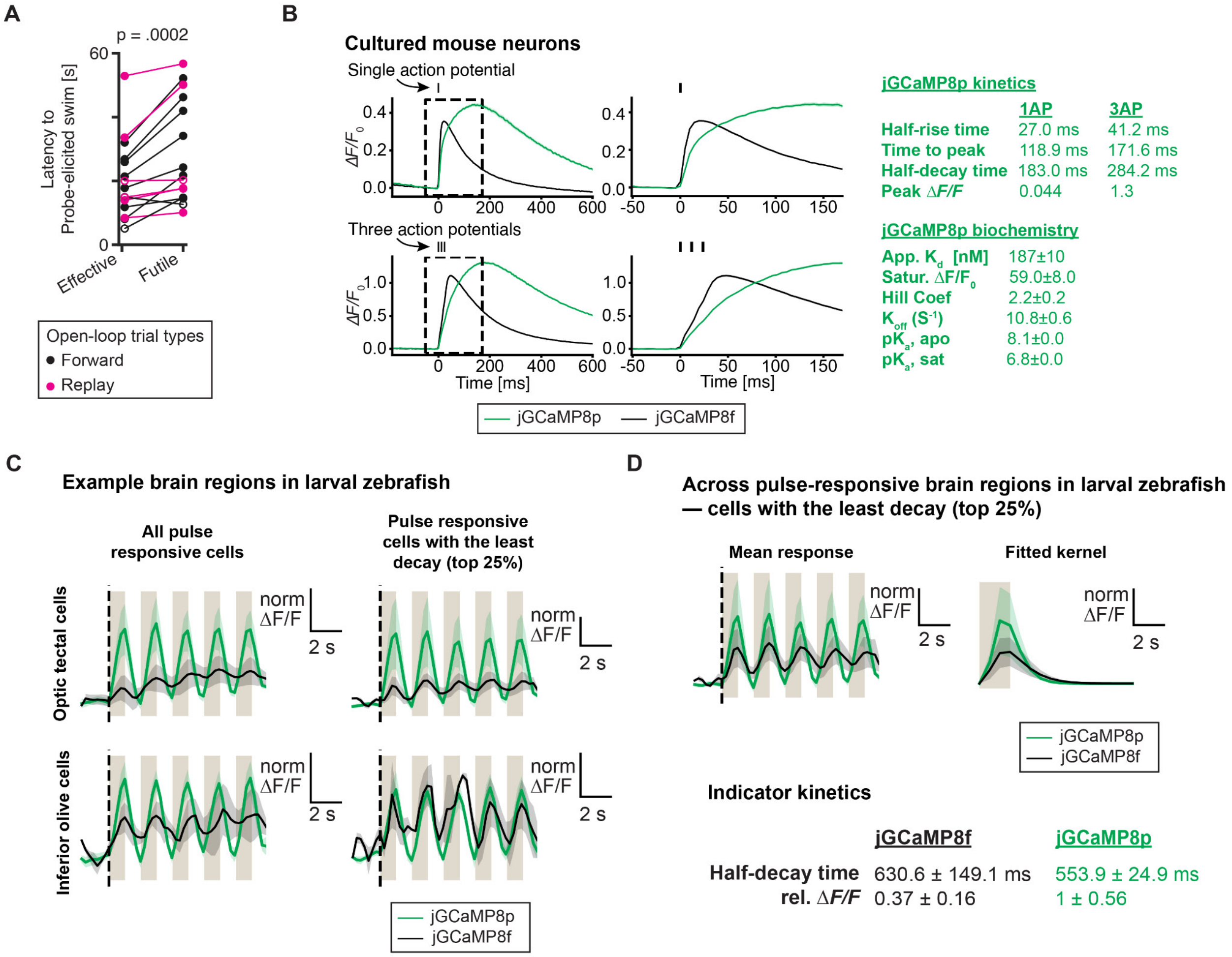

Figure S1. Related to Fig. 1. Statistics of fish behaviors in Effective and Futile trials, and calcium indicator kinetics.

(A) Support material for Fig. 1G, with Futile trials broken down into two types – black: fish experienced $G_{ms}=0$ and v_{stim} is a constant in Futile trials; magenta: fish experienced $G_{ms}=0$ and v_{stim} is a replay from a previous Effective trial, i.e. open-loop replay, in Futile trials. Probe-evoked swims were delayed in Futile trials, ***p=0.0002, N=15 fish; p=0.0078, N=8 fish in constant-forward-grating Futile trials; p=0.0156, N=7 fish in open-loop-replay Futile trials; paired signed-rank tests.

(B) Fluorescence responses of jGCaMP8p-expressing cultured mouse neurons to one (top) and three (bottom) action potentials in response to electric field stimulation in comparison to jGCaMP8f.[65] Middle column, zoomed-in from the left (dash-boxed) to highlight rise kinetics (middle). Solid lines, mean; shaded areas, SEM; jGCaMP8p, green N=940 neurons, 7 plates, 40 wells; jGCaMP8f, black, N=804 neurons, 5 plates, 54 wells).

bioRxiv preprint doi: https://doi.org/10.64898/2026.02.05.704034; this version posted February 5, 2026. The copyright holder for this preprint (which was not certified by peer review) is the author/funder, who has granted bioRxiv a license to display the preprint in perpetuity. It is made available under aCC-BY 4.0 International license.

Right, a summary table of jGCaMP8p kinetics.

(C) *In vivo* fluorescence responses of all (left) and the fastest (right) neurons in the optic tecum (top) and inferior olive (bottom) expressing either jGCaMP8f (black) or jGCaMP8p (green) while fish is in the Disengaged state, aligned to the start of the Probe epoch (dotted line). Solid lines, mean; shaded areas, SEM across fish (jGCaMP8p, green, N = 4 fish; jGCaMP8f, black, N = 3 fish). Khaki bars, visual motion pulses. Vertical scale bar, normalized ΔF/F, representing that of the indicator with the larger ΔF/F, jGCaMP8p, for comparison.

(D) *In vivo* fluorescence responses of sensory neurons with the least amount of motion integration, to isolate GCaMP8f and GCaMP8p kinetics for neuronal integration. Fish expressed either jGCaMP8f (black) or jGCaMP8p (green) and were in Futile trials. Left, responses to a series of visual motion pulses aligned to the onset of the Probe epoch (dotted lines). Right, the fitted impulse response to a single motion pulse. Khaki bars, visual motion pulses. Bottom, characterization of the kinetics of the impulse response. ΔF/F is normalized by that of the indicator with the larger ΔF/F, jGCaMP8p, for comparison. Note that in larval zebrafish at room temperature, jGCaMP8p exhibits faster kinetics than jGCaMP8f, whereas in mouse neuron culture jGCaMP8f exhibits faster kinetics, suggesting species/temperature differences. Due to GCaMP8p's faster kinetics in zebrafish, this is the indicator used for most experiments in this work.

bioRxiv preprint doi: https://doi.org/10.64898/2026.02.05.704034; this version posted February 5, 2026. The copyright holder for this preprint (which was not certified by peer review) is the author/funder, who has granted bioRxiv a license to display the preprint in perpetuity. It is made available under aCC-BY 4.0 International license.

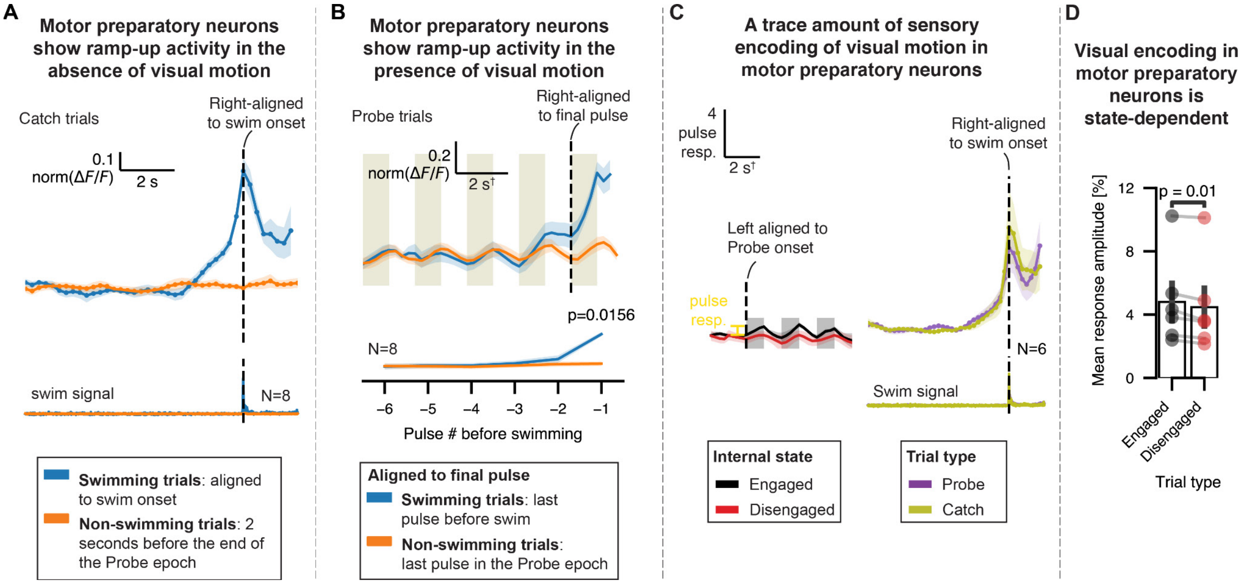

Figure S2. Related to Fig. 2E. Dynamics of motor preparatory neurons.

(A) Motor preparatory neurons ramp up prior to swimming in Catch trials. Top, average neuronal activity ramps up from ~3 s before swimming (blue; swimming trials); this ramping dynamics was not present in non-swimming trials (orange) (mean±sem across N=8 fish; min-max normalized to the lowest and highest activity values of the activity trace on swimming trials). Bottom, example swim signal from N=8 fish.

(B) Motor preparatory neurons also exhibit weak visual motion response in Probe trials. Motor preparatory neurons displayed increased activity right before swimming. Top, the same convention as (A), except aligned to the last pulse before swimming, with traces min-max normalized to average activity trace of swimming trials. Khaki bars, visual motion pulses. Bottom, average neuronal response to visual pulses; line and shaded area, mean ± sem across N=8 fish; min-max normalized to the lowest and highest response; two-tailed Wilcoxon signed-rank test.

(C) Motor preparatory neurons exhibit state-dependent visual response and ramp prior to swimming. Left, early-phase motor preparatory neuronal activity aligned to the onset of Probe epoch. Black, Engaged state; red, Disengaged state. Right, motor preparatory neuronal activity aligned to swimming; purple, Probe trials; olive, Catch trials. Shaded area, SEM across N=6 fish (two fish with insufficient state-related trials were excluded).

(D) Early-phase visual response in motor preparatory neurons is state-dependent. Bar plots of the neural activity in the first 3 pulses of the Probe epoch in Engaged and Disengaged states; error bars, SEM across N=6 fish; two-tailed Wilcoxon signed-rank test.

Across panels, s† means 1 second for all but one fish; for one fish the visual motion pulses were 2 s long, interspersed by 2 s, in which case the time traces were scaled so that s† represents 2 seconds to enable population averaging.

bioRxiv preprint doi: https://doi.org/10.64898/2026.02.05.704034; this version posted February 5, 2026. The copyright holder for this preprint (which was not certified by peer review) is the author/funder, who has granted bioRxiv a license to display the preprint in perpetuity. It is made available under aCC-BY 4.0 International license.

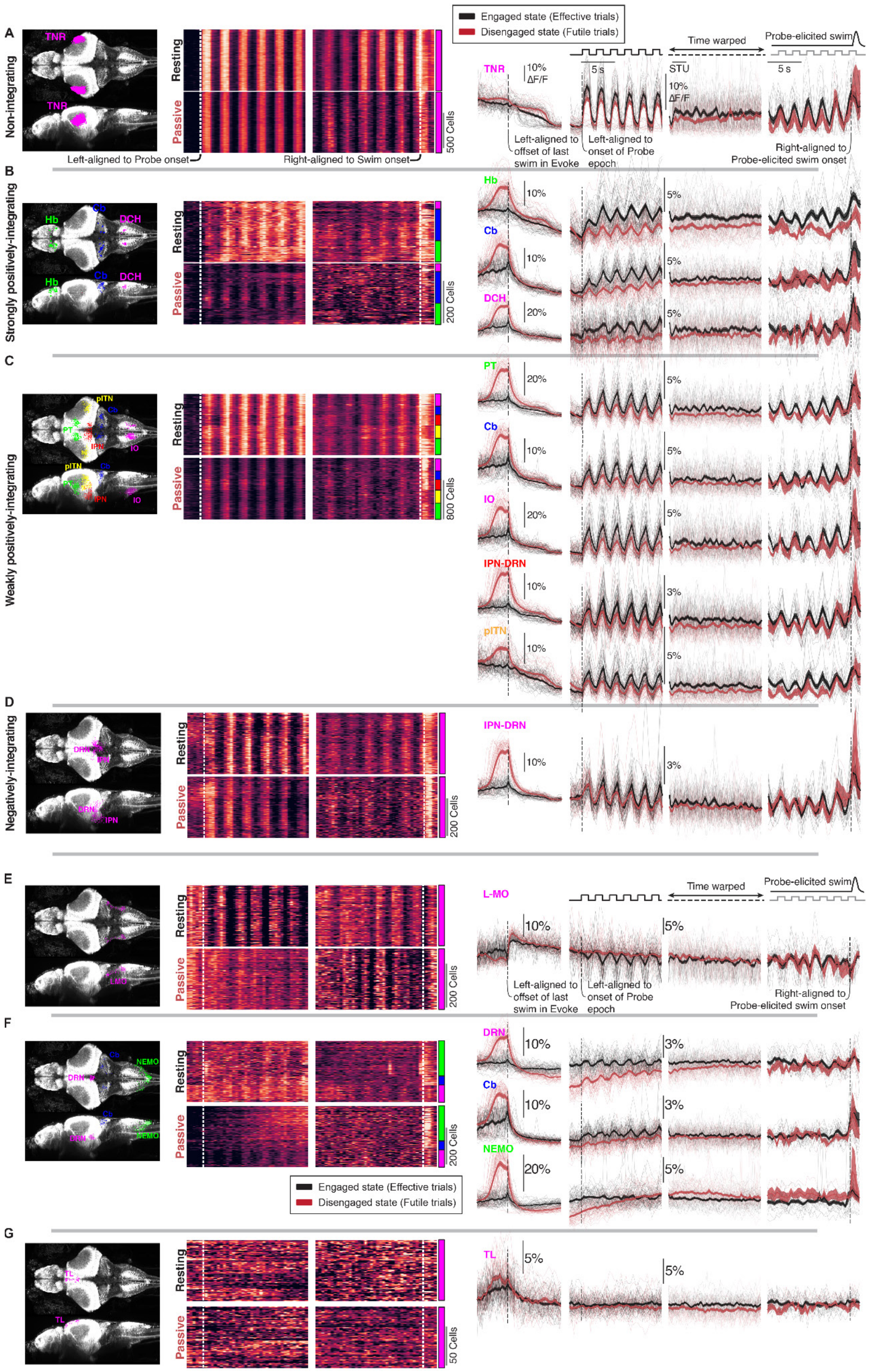

Figure S3. Related to Fig. 3. Multiple sensory regions are modulated during the transition into and by the Disengaged state in different ways.

Details of the spatiotemporal clustering algorithm: state-modulated visual motion responsive cells (in a representative fish) are subdivided into groups of regions based on their integrative properties using hierarchical clustering of neural responses to the pulse

bioRxiv preprint doi: https://doi.org/10.64898/2026.02.05.704034; this version posted February 5, 2026. The copyright holder for this preprint (which was not certified by peer review) is the author/funder, who has granted bioRxiv a license to display the preprint in perpetuity. It is made available under aCC-BY 4.0 International license.

series (a concatenated vector of time series of the neural activity in first 6 pulses in Effective and Futile trials;rows) and then by anatomical regions (DBSCAN subclustering[53] based on neuronal spatial locations, with maximum distance between two neurons in the cluster <30 μm; sub-rows). The location of the anatomical regions in each group can be found in the brain maps (first column). Their single-cell activity can be found in heatmaps aligned to the onset of the Probe epoch (second column) and Probe-evoked swim (third column). Single-cell activity for both the Engaged and Disengaged states are shown. Single region activity is represented as traces aligned to the offset of swimming in the Evoke epoch (fourth column), aligned to the start of the Probe epoch (fifth column), aligned to both the start of the Probe epoch and onset of the probe-evoked swim (STU: scaled time unit) (sixth column), and aligned to the onset of the probe-evoked swim (seventh column). Single region activity in both the Engaged (black traces) and Disengaged (red traces) states are shown. Thin lines, neural dynamics on single trials; thick line,trial average; shaded area, SEM across trial.

(A) The tectal neuropil region (TNR) is a visual-motion responsive region that does not integrate over pulses. The tectal neuropil has activity that is generally higher in the Evoke and Probe epochs, but that activity eventually converges between trial types before the onset of the Probe epoch. Activity during the Probe epoch is suppressed when the fish is in the Disengaged state, a trend that continues until the fish is about to swim.

(B) The habenula (Hb), cerebellum (Cb) and dorsocaudal hindbrain (DCH) regions contain cells that strongly integrate over the visual motion pulses. All three regions are more strongly activated during the Evoke epoch before the fish enters the Disengaged state. This difference converges during the Pause epoch (note that this is a subset of the dorsocaudal hindbrain region that was clustered together with the other cell groups; other cells in this region show more persistent activity; Fig. S6). Activity during the Probe epoch is suppressed when the fish is in the Disengaged state. Activity in the habenula does not seem to converge between trial types, even after the fish performs the probe-evoked swim. On the other hand, activity in Cb and DCH regions seem to converge earlier.

(C) The pretectum (PT), cerebellum (Cb), inferior olive (IO), interpeduncular nucleus / dorsal raphe nucleus (IPN-DRN), posterior lateral tectal neuropil region (pITN) contain cells that weakly integrate over the visual motion pulses. All regions are more strongly activated during the Evoke epoch before the fish enters the Disengaged state. This difference converges between trial types during the Pause epoch. Activity during the Probe epoch is suppressed when fish is in the Disengaged state and become increasingly less so during the course of the Probe epoch. Activity seems to have converged (i.e., became similar between trial types, but typically at a later time in Futile trials) before the onset of the probe-evoked swim.

(D) The interpeduncular nucleus / dorsal raphe nucleus (IPN-DRN) region also contains cells that integrate negatively over the visual motion pulses. These cells are activated

bioRxiv preprint doi: https://doi.org/10.64898/2026.02.05.704034; this version posted February 5, 2026. The copyright holder for this preprint (which was not certified by peer review) is the author/funder, who has granted bioRxiv a license to display the preprint in perpetuity. It is made available under aCC-BY 4.0 International license.

more strongly during the Evoke epoch before the fish enters the Disengaged state, a difference that converges during the Pause epoch. The visual motion pulses are encoded very similarly between the Engaged and Disengaged states.

(E) The lateral medulla oblongata (L-MO) contains cells that encode the visual motion pulses negatively. Activity during the Evoke epoch is not very different between the Engaged and Disengaged states. However, the encoding of visual motion is suppressed to a large degree when the fish is in the Disengaged state. This response eventually recovers, but not until it gets closer to the Probe-evoked swim (note, this happens on average at later times in Futile trials).

(F) The dorsal raphe nucleus (DRN), cerebellum (Cb) and NE-MO contains neurons that are much more highly activated during the Evoke epoch before the fish enters the Disengaged state. While that difference converges during the Pause epoch, presumably after the fish enters the Disengaged state, it eventually dips lower than that of the Engaged state. Much of this visual motion - independent difference is maintained until right before the fish performs the probe-evoked swim.

(G) The torus longitudinalis (TL) is a region that does not respond to the visual motion stimuli but shows a difference in activity between the Engaged and Disengaged states. Specifically, activity is slightly suppressed when fish is in the Disengaged state, most clearly seen in the time warped traces.

bioRxiv preprint doi: https://doi.org/10.64898/2026.02.05.704034; this version posted February 5, 2026. The copyright holder for this preprint (which was not certified by peer review) is the author/funder, who has granted bioRxiv a license to display the preprint in perpetuity. It is made available under aCC-BY 4.0 International license.

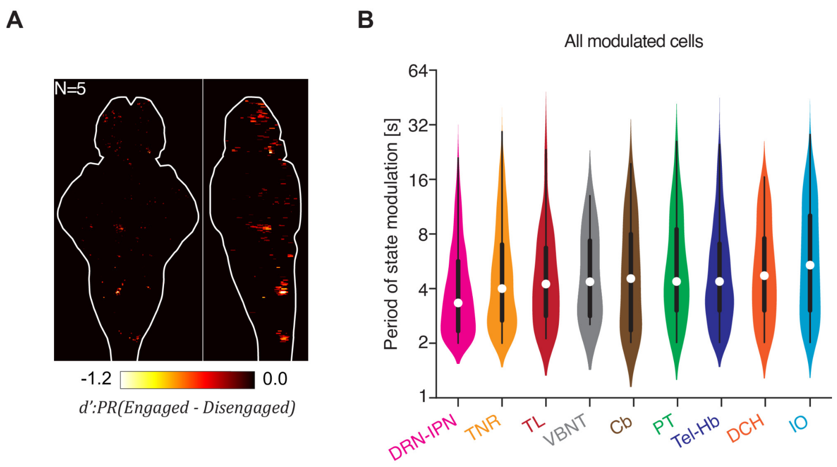

Figure S4. Related to Fig. 3. Additional analyses.

(A) Support material for Fig. 3C. Brain map showing cells with a greater response in the Disengaged state than in the Engaged state, as quantified by the discriminability index (d'), with negative values here for comparison to positive values shown in Fig. 3C. Only 3.19 ± 2.01% of the pulse-responding neurons were negatively modulated; N=5 fish, mean ± SD.

(B) Support material for Fig. 3G. Brain regions contain cells that are modulated for a large range of durations, from 0.7 sec to 42 sec. Violin plots showing the distribution of modulation duration across cells and across fish. A miniature box-and-whisker plot showing the quartiles of the data is also shown.

bioRxiv preprint doi: https://doi.org/10.64898/2026.02.05.704034; this version posted February 5, 2026. The copyright holder for this preprint (which was not certified by peer review) is the author/funder, who has granted bioRxiv a license to display the preprint in perpetuity. It is made available under aCC-BY 4.0 International license.

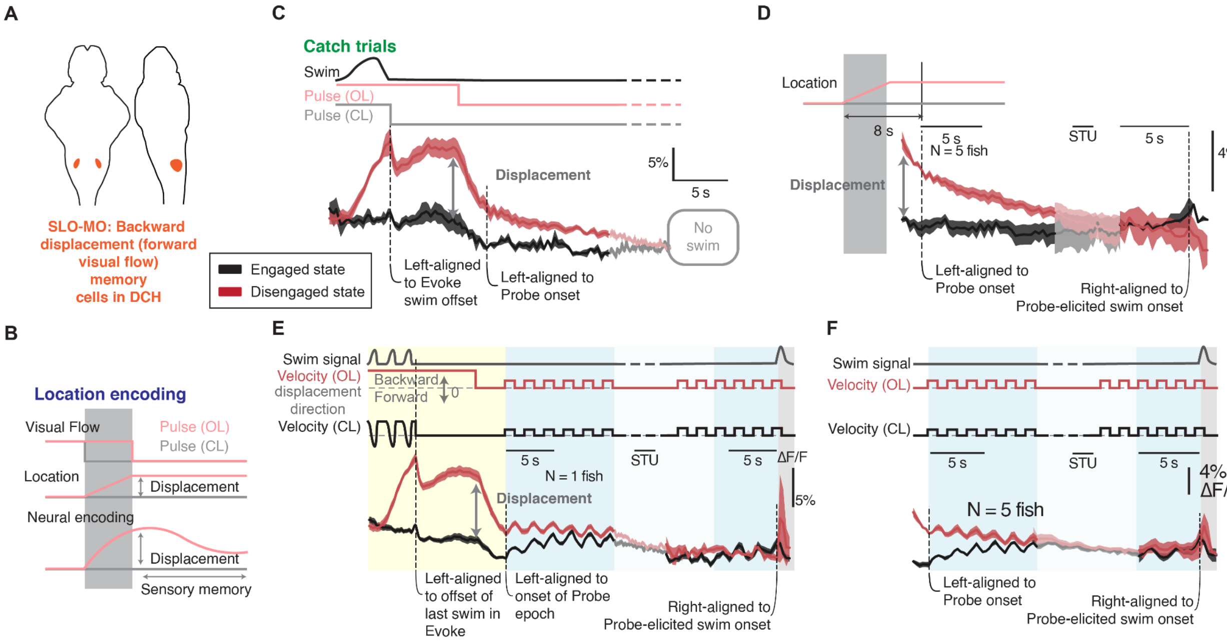

Figure S5. Related to Fig. 3. Location-encoding in SLO-MO dynamics.

Due to the experimental design, backward displacement-encoding SLO-MO neurons showed higher activity in Futile trials, reflecting the additional ≥5 seconds of forward-grating exposure during the Evoke epoch. This finding is consistent with their proposed role in sensory integration and displacement memory.[41]

(A) Brain loci of the backward displacement-encoding cells in SLO-MO.

(B) Schematics of visual flows and displacements during Effective (gray) and Futile (pink) types, where fish were exposed to forward-grating motion for additional 5 seconds during the Futile Evoke epoch, and the hypothesized neural encoding of displacement in SLO-MO.

(C-F) SLO-MO activity is sensory history dependent and the difference of neural dynamics in Effective and Futile trials reflects the difference in displacements; black, Effective trials; red, Futile trials. **(C,D)** Average SLO-MO dynamics in Catch trials (no motion pulse during 'Probe' epoch). **(C)** Single fish; shaded area, SEM across trials. **(D)** Multiple fish; shaded area, SEM across fish, N=5. **(E,F)** the same as **(C,D)** in Probe trials. STU: scaled time unit.

bioRxiv preprint doi: https://doi.org/10.64898/2026.02.05.704034; this version posted February 5, 2026. The copyright holder for this preprint (which was not certified by peer review) is the author/funder, who has granted bioRxiv a license to display the preprint in perpetuity. It is made available under aCC-BY 4.0 International license.

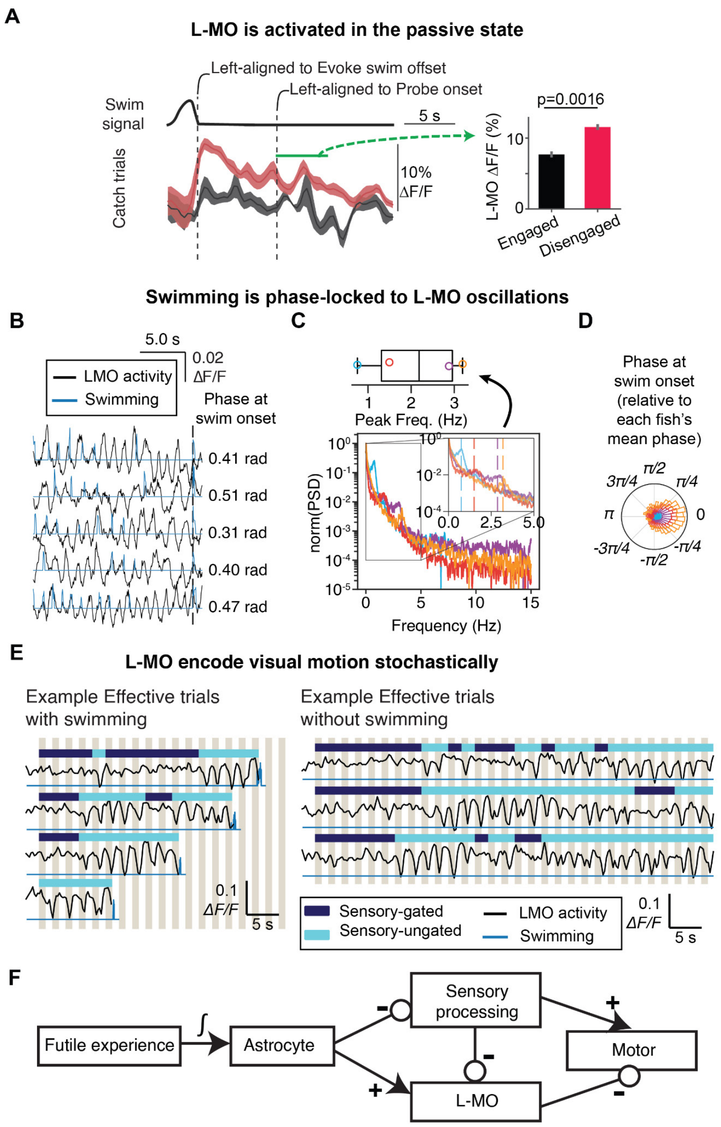

Figure S6. Related to Fig. 4. L-MO dynamics.

(A) State-dependent L-MO dynamics in Catch trials. Left, neural dynamics aligned to the last swim in the Evoke epoch. Black, Effective trials; red, Futile trials; shaded area, SEM across fish, N=5. Right, Bar plots of neural dynamics in the early phase of Catch trials. Error bar, SD across neurons; p = 0.0016, paired signed-rank test across neurons. Previous study showed L-MO were excited during astroglial activation. We tested whether L-MO were excited in Futile catch trials.

(B) Swimming is phase-locked to L-MO oscillations. Black, L-MO activity; blue, swim signal. Swimming phases to L-MO oscillations were shown for 5 example swim bouts on the right; vertical dotted line, swim onset.

(C) L-MO oscillated at 0.5-3 Hz. Bottom, power spectral density of L-MO oscillations. Inset, zoomed in at <5 Hz. Top, box plot of peak frequencies of L-MO oscillations across

bioRxiv preprint doi: https://doi.org/10.64898/2026.02.05.704034; this version posted February 5, 2026. The copyright holder for this preprint (which was not certified by peer review) is the author/funder, who has granted bioRxiv a license to display the preprint in perpetuity. It is made available under aCC-BY 4.0 International license.

N = 4 fish. Color, fish identity.

(D) Rose plot of the histograms of swimming phase to L-MO oscillations across N=4 fish. Color, fish identity.

(E) L-MO stochastically responds (navy, no response; light blue, response to visual motion) to visual motion (khaki bars) with decreased calcium in swimming trials (left) and non-swimming trials (right); response becomes robust before swim; black line, ΔF/F trace; blue line, swim trace.

(F) Hypothesized model of astrocytic modulation of sensory processing and L-MO. Astrocytic calcium integrates futile swims. Previous work showed that astrocytes activate GABAergic neurons in area L-MO, a motor-suppression hub, thereby inhibiting swimming during the passive state.[27,37] Reduced L-MO activity may relieve this suppression and facilitate swimming (Fig. 4F). This study shows that astrocytes also dampen sensory processing and integration (Fig. 5). In addition, forward visual motion inhibits L-MO activity (Fig. 4G, Fig. S6E), producing state-dependent disinhibition of L-MO when astrocytes are activated. Thus, astrocytic modulation of L-MO synergizes with their suppression of visual processing to reconfigure sensorimotor transformations during state switches.

bioRxiv preprint doi: https://doi.org/10.64898/2026.02.05.704034; this version posted February 5, 2026. The copyright holder for this preprint (which was not certified by peer review) is the author/funder, who has granted bioRxiv a license to display the preprint in perpetuity. It is made available under aCC-BY 4.0 International license.

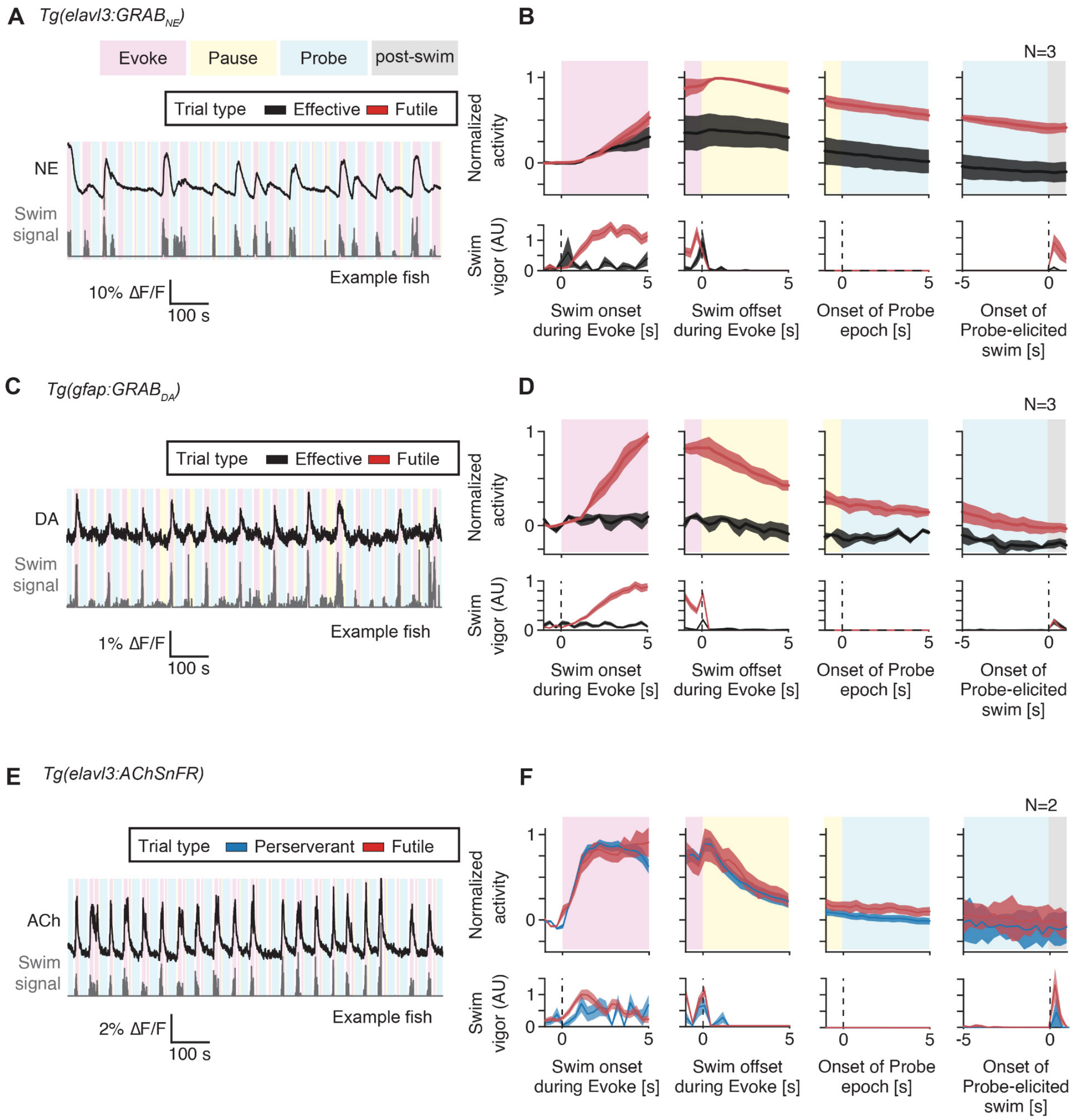

Figure S7. Related to Fig. 5. Futile swimming is associated with norepinephrine, dopamine and acetylcholine release.

(A,B) Brainwide norepinephrine signals during trials. **(A)** Whole-brain averages show that norepinephrine levels rise sharply during strong futile swimming during the open-loop evoke in Futile trials. **(B)** Whole-brain averages show that norepinephrine is released to a large extent when the fish underwent strong futile swimming before entering the Disengaged state, with levels remaining elevated after the offset of swimming. Note that slow indicator dynamics at room temperature in zebrafish may contribute to the long time constants. Black, Effective trials; red, Futile trials. Shaded area, SEM across N=3 fish.

(C,D) Brainwide dopamine release, conventions as in (A). Note that indicator dynamics at room temperature in zebrafish may contribute to observed slow dynamics. N=3 fish.

(E,F) Acetylcholine release, depicted as in (A,B) but during Futile and Perseverant

bioRxiv preprint doi: https://doi.org/10.64898/2026.02.05.704034; this version posted February 5, 2026. The copyright holder for this preprint (which was not certified by peer review) is the author/funder, who has granted bioRxiv a license to display the preprint in perpetuity. It is made available under aCC-BY 4.0 International license.

behavior, where fish underwent futile swimming but have yet to 'give up'. The same comment about potentially slow indicator dynamics applies. N=2 fish.
